# Codominant IgG and IgA expression with minimal vaccine mRNA in milk of BNT162b2 vaccinees

**DOI:** 10.1038/s41541-021-00370-z

**Published:** 2021-08-19

**Authors:** Jia Ming Low, Yue Gu, Melissa Shu Feng Ng, Zubair Amin, Le Ye Lee, Yvonne Peng Mei Ng, Bhuvaneshwari D/O Shunmuganathan, Yuxi Niu, Rashi Gupta, Paul Anantharajah Tambyah, Paul A. MacAry, Liang Wei Wang, Youjia Zhong

**Affiliations:** 1 Department of Paediatrics, Yong Loo Lin School of Medicine, National University of Singapore, Singapore, Singapore; 2 Department of Neonatology, Khoo Teck Puat—National University Children’s Medical Institute, National University Health System, Singapore, Singapore; 3 Antibody Engineering Programme, Life Sciences Institute, National University of Singapore, Singapore, Singapore; 4 Department of Microbiology and Immunology, Yong Loo Lin School of Medicine, National University of Singapore, Singapore, Singapore; 5 Singapore Immunology Network, Agency for Science, Technology and Research, Singapore, Singapore; 6 Infectious Diseases Translational Research Programme, Department of Medicine, Yong Loo Lin School of Medicine, National University of Singapore, Singapore, Singapore; 7 Khoo Teck Puat—National University Children’s Medical Institute, National University Health System, Singapore, Singapore; 8 Duke-NUS Medical School, Singapore, Singapore

**Keywords:** Viral infection, Outcomes research

## Abstract

Lactating women can produce protective antibodies in their milk after vaccination, which has informed antenatal vaccination programs for diseases such as influenza and pertussis. However, whether SARS-CoV-2-specific antibodies are produced in human milk as a result of COVID-19 vaccination is still unclear. In this study, we show that lactating mothers who received the BNT162b2 vaccine secreted SARS-CoV-2-specific IgA and IgG antibodies into milk, with the most significant increase at 3–7 days post-dose 2. Virus-specific IgG titers were stable out to 4–6 weeks after dose 2. In contrast, SARS-CoV-2-specific IgA levels showed substantial decay. Vaccine mRNA was detected in few milk samples (maximum of 2 ng/ml), indicative of minimal transfer. Additionally, infants who consumed post-vaccination human milk had no reported adverse effects up to 28 days post-ingestion. Our results define the safety and efficacy profiles of the vaccine in this demographic and provide initial evidence for protective immunity conferred by milk-borne SARS-CoV-2-specific antibodies. Taken together, our study supports recommendations for uninterrupted breastfeeding subsequent to mRNA vaccination against COVID-19.

## Introduction

Lactating women who have recovered from respiratory virus infections produce antibodies in human milk that can neutralize offending viruses in vivo^1^. The production of protective antibodies in human milk after influenza vaccination in lactating women confers local mucosal immunity to infants and has informed antenatal vaccination programs for diseases such as influenza and pertussis in lactating women^2,3^.

However, similar evidence for coronavirus disease of 2019 (COVID-19) messenger ribonucleic acid (mRNA) vaccines is scarce^4^. As a result, vaccine advisories from various health authorities have been cautious in recommending vaccinations for lactating women. The American College of Obstetricians and Gynecologists (ACOG) and the Royal College of Obstetricians and Gynecologists (RCOG, UK) both state that COVID-19 vaccines may be offered to lactating women, while acknowledging that adequate safety data are not available^5,6^. In countries such as Singapore, women had been advised to express and discard human milk for up to 7 days after vaccination^7^. However, such measures may disrupt mother–child bonding and may result in premature cessation of breastfeeding^8^.

There is emerging evidence that SARS-CoV-2-specific antibodies are detectable in human milk post-vaccination^9–12^; however, the amount of SARS-CoV-2-specific immunoglobulin G (IgG) and immunoglobulin A (IgA) have not been clearly quantified. It is also unknown whether vaccine components such as mRNA are transferred in human milk; preliminary studies purportedly detect no vaccine mRNA in human milk^13^.

Our aims are (1) to longitudinally quantify SARS-CoV-2-specific IgA and IgG in human milk of lactating women who received COVID-19 mRNA vaccine, with reference to a cohort convalescent from antenatal COVID-19 as well as a control cohort of healthy lactating women, and (2) to detect and quantify vaccine mRNA in human milk after vaccination.

We hypothesize that BNT162b2, an mRNA vaccine encoding the immunogenic SARS-CoV-2 spike protein, has minimal leakage into human milk after vaccination, and induces the production and secretion of spike- and receptor-binding domain (RBD)-specific IgA and IgG into human milk in a durable manner.

## Results

### Maternal and infant characteristics

Fourteen lactating healthcare workers were recruited; all received two doses of the BNT162b2 (Pfizer/BioNtech) vaccine, with the second dose given on day 21. The women were of a mean age of 33.2 [standard deviation, SD 3.6] years of age, ten (71%) were Chinese and four (29%) were Malay. At recruitment, the women were a mean of 9.0 [SD 3.8] months postpartum (Table 1). Subjects were sampled at five time points: pre-vaccination (T1), 1–3 days after dose 1 (T2), 7–10 days after dose 1 (T3), 3–7 days after dose 2 of COVID-19 mRNA vaccine (T4), and 4–6 weeks after dose 2 of COVID-19 mRNA vaccine (T5).five time points: pre-vaccination (T1), 1–3 days after dose 1 (T2), 7–10 days after dose 1 (T3), 3–7 days after dose 2 of COVID-19 mRNA vaccine (T4), and 4–6 weeks after dose 2 of COVID-19 mRNA vaccine (T5). All subjects had samples available up to T4, and ten subjects had samples available up to T5; a total of 66 samples were collected and analyzed. All infants were born full term and healthy. No woman or infant experienced any serious adverse event during the 28-day study period. None of the women had mastitis after vaccination. Twelve of the 14 infants were breastfed within 72 h after their mothers were vaccinated; two infants were not fed breast milk within 72 h of vaccination. None of these 12 developed any adverse reactions—including fever, rash, vomiting, diarrhea, cough or rhinorrhoea—up to 28 days after ingestion of post-vaccination human milk.

**Table 1:** Clinical and demographic features of vaccinated cohort, convalescent cohort, and healthy cohort of women.

	Vaccinated cohort (*n* = 14)	Convalescent cohort (*n* = 6)	Healthy cohort (*n* = 9)
Age, mean [standard deviation, SD], years	33.2 [3.6]	30.8 [4.1]	32.1 [4.1]
Ethnicity	10 Chinese 4 Malay	1 Chinese 2 Malay 1 Indian 2 Caucasian	6 Chinese 1 Malay 2 Indian
Chronic diseases	13 None 1 Thalassemia minor	5 None 1 Pre-existing hepatitis C	8 None 1 Pre-existing hepatitis C
Antenatal conditions	14 None	6 None	9 None
Smoking history	14 No	5 No 1 Smoker	8 No 1 Smoker
Types of feeds provided	13 Exclusively breastfed 1 Mixed feeds	4 Exclusive breastfeeding 2 Mixed feeding	6 Exclusive breastfeeding 3 Mixed feeding
Months postpartum, mean [SD]	9.0 [3.8]	NA	NA

Women in the convalescent cohort had COVID-19 with a mean of 136 [SD 80] days before delivery; thus, human milk collected 1 month postpartum was about 5.5 months from the point of initial antigenic exposure. Subjects in the convalescent and healthy women did not receive SARS-CoV-2 vaccination during the study. Clinical details of the convalescent and healthy women in the reference cohort are available in Table 1, and clinical details of their infants are available in Supplementary Tables 1 and 2.

### Levels of SARS-CoV-2-specific IgA and IgG in human milk of lactating women

**Fig. 1:**
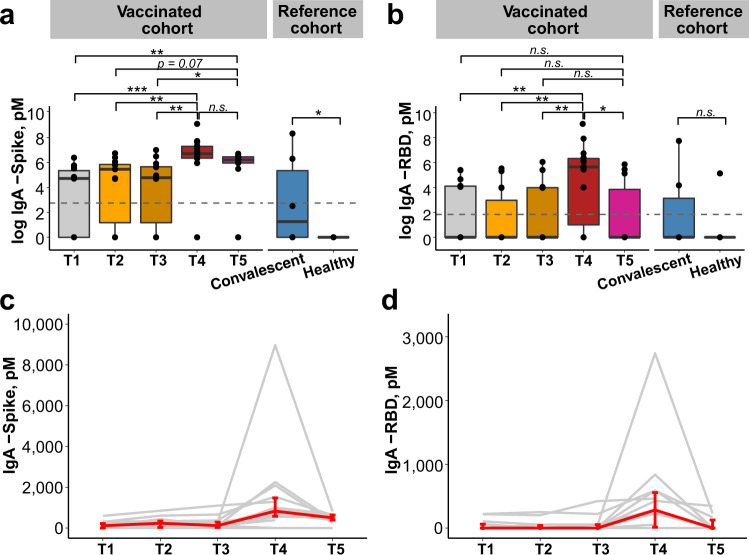
SARS-CoV-2-specific IgA antibodies in human milk samples. Concentration [pM^a^] of IgA antibodies against spike (**a**) and RBD (**b**) in human milk for vaccinated women (*n* = 14) across T1–T4, (*n* = 10) at T5; convalescent (*n* = 6) and healthy women (*n* = 9) used as reference cohort. Gray dotted lines reflect the limit of assay detection. *p* values were calculated with Kruskal–Wallis test and Dunn’s post hoc test, *p < 0.05, **p < 0.01, ***p < 0.001, n.s. non-significant. Box and whiskers plots show median (center line), interquartile range (box), and 10th and 90th percentiles (whiskers). Increase in IgA antibodies against spike (**c**) and RBD (**d**) over time. Line plots show the median, and error bars show the first and third quartiles. Each line in gray represents data from one individual and the median ± IQR is represented in red. RBD receptor-binding domain, IgA immunoglobulin A, pM picomolar, IQR interquartile range, T1 pre-vaccination, T2 1–3 days after dose 1 of BNT162b2 vaccine, T3 7–10 days after dose 1 of BNT162b2 vaccine, T4 3–7 days after dose 2 of BNT162b2 vaccine, T5 4–6 weeks after dose 2 of BNT162b2 vaccine. ^a^SI conversion factors: to convert concentration from pM to M, multiply values by 10^12^.

A human monoclonal antibody binding to SARS-CoV-2 was recombinantly engineered and expressed as SARS-CoV-2-specific human IgG and human IgA. This resulted in the production of monoclonal antibodies of different isotypes with the same antigen-binding ability, which were utilized as reference reagents for construction of standard curves for the quantitative ELISA (Supplementary Fig. 1). Human milk samples were evaluated for SARS-CoV-2-specific IgA binding reactivity against SARS-CoV-2 spike and receptor binding domain (RBD) (Fig. 1 and Supplementary Fig. 2).

Vaccination induced a strong SARS-CoV-2-specific IgA response at T4 (i.e., 3–7 days after dose 2 of COVID-19 mRNA vaccine). Human milk samples from vaccinated women from T4 had medians of 827 picomolar (pM) of anti-spike (Fig. 1a and Supplementary Spreadsheets 1 and 2) and 282 pM of anti-RBD IgA (Fig. 1b and Supplementary Spreadsheets 1 and 2), both significantly higher than the concentrations from earlier time points (*p* < 0.001). Human milk samples evaluated at T4 also exhibited significantly higher levels of SARS-CoV-2-specific IgA compared to the reference samples provided by convalescent women who had antenatal COVID-19. IgA was not detected in one woman at all timepoints, including T4 and T5 (Fig. 1c, d and Supplementary Fig. 2). At T5 (i.e., 4–6 weeks after dose 2 of COVID-19 mRNA vaccine), a reduction was observed in the anti-spike (median: 499 pM) and the anti-RBD (median: 0 pM) IgA response (Fig. 1a, b; Supplementary Fig. 2; and Supplementary Table 2).

**Fig. 2:**
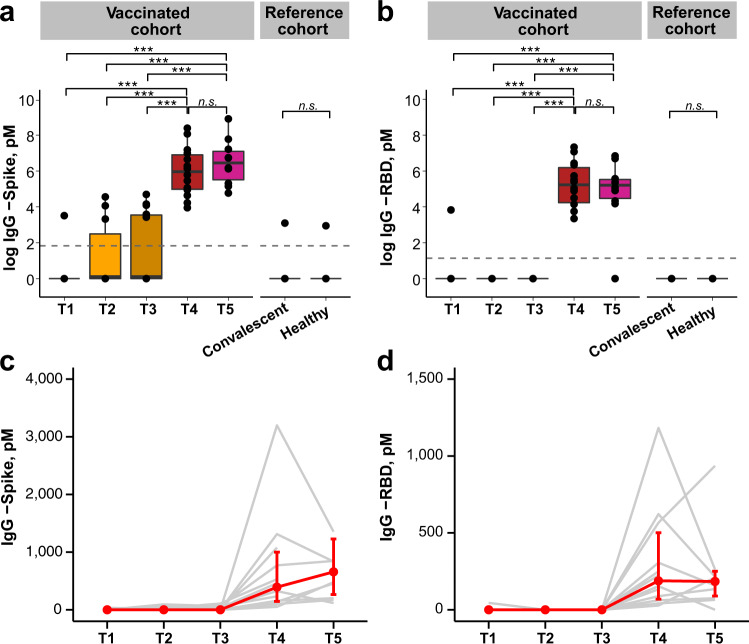
SARS-CoV-2-specific IgG antibodies in human milk samples. Concentration [pM^a^] of IgG antibodies against spike (**a**) and RBD (**b**) in human milk for vaccinated women (*n* = 14) across timepoints T1–T4, with (*n* = 10) at T5; convalescent (*n* = 6) and healthy women (*n* = 9) used as reference cohort. Gray dotted lines reflect the limit of assay detection. *p* values were calculated with Kruskal–Wallis test and Dunn’s post hoc test, *p < 0.05, **p < 0.01, ***p < 0.001, n.s. non-significant. Box and whiskers plots show median (center line), interquartile range (box), and 10th and 90th percentiles (whiskers). Increase in IgG antibodies against spike (**c**) and RBD (**d**) over time. Line plots show the median, and error bars show the first and third quartiles. Each line in gray represents data from one individual and the median ± IQR is represented in red. RBD receptor-binding domain, IgG immunoglobulin G, pM picomolar, IQR interquartile range, T1 pre-vaccination, T2 1–3 days after dose 1 of BNT162b2 vaccine, T3 7–10 days after dose 1 of BNT162b2 vaccine, T4 3–7 days after dose 2 of BNT162b2 vaccine, T5 4–6 weeks after dose 2 of BNT162b2 vaccine. ^a^SI conversion factors: to convert concentration from pM to M, multiply values by 10^12^.

Given the robustness of the SARS-CoV-2-specific IgA response, and that SARS-CoV-2-specific IgG has been shown to be present in human milk^9–12^, we next assessed the concentration of IgG targeting spike and RBD in human milk samples collected at all timepoints. The median concentrations of anti-spike and anti-RBD IgG at T4 were 392 and 188 pM (Supplementary Table 2), respectively. In contrast to the specific IgA, these IgG levels remained high at T5, with median concentrations of 657 pM anti-spike IgG and 184 pM anti-RBD IgG (Supplementary Table 2). These levels at T4 and T5 were significantly higher compared to the concentration at T1 (*p* < 0.001), and to the convalescent cohort, all of which were negligible (Fig. 2a, b; Supplementary Fig. 3; and Supplementary Spreadsheet 1). There was no SARS-CoV-2-specific IgA or IgG detected in healthy unvaccinated lactating women. An increase in SARS-CoV-2-specific IgG in human milk samples were observed in all human milk samples (14/14) after the second dose of the mRNA vaccine at T4 (Fig. 2c, d and Supplementary Fig. 3).

At T4 and T5, both SARS-CoV-2-specific IgA and IgG antibodies were present in human milk. The ratio of SARS-CoV-2-specific IgA to IgG was calculated. A ratio of more than 1 reflects more SARS-CoV-2-specific IgA present than SARS-CoV-2-specific IgG. Conversely, a ratio of <1 reflects more SARS-CoV-2-specific IgG than IgA.

Optical density at 450 nanometer (OD_450_) is a raw assay readout for quantitative ELISA that is highly dependent on factors such as the sensitivity of the test kit. Utilizing this raw assay readout, OD_450_ values of SARS-CoV-2-specific IgG were observed to be significantly above that of SARS-CoV-2-specific IgA at both T4 and T5, with 79% (11/14) T4 samples and 100% (10/10) T5 samples following this trend (Supplementary Fig. 4).

**Fig. 3:**
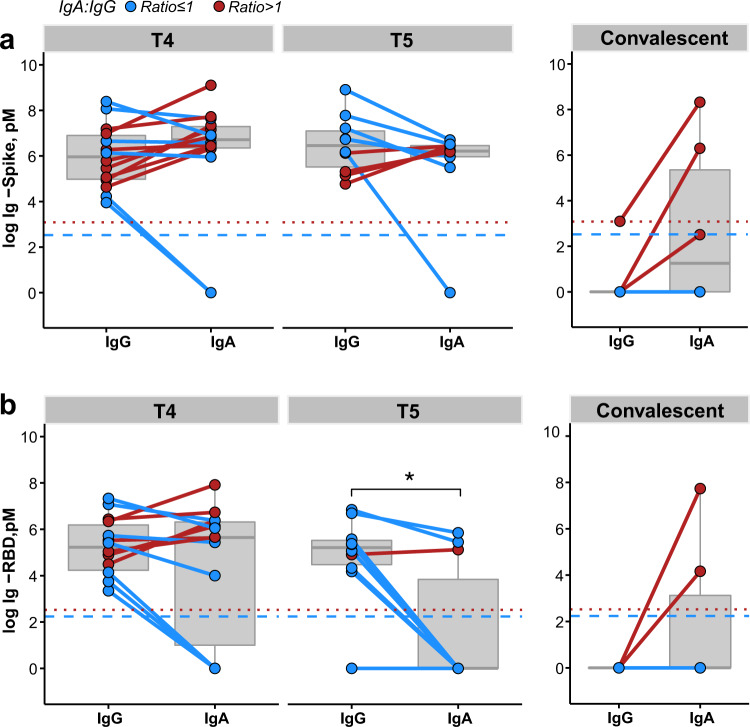
Vaccinated women have both SARS-CoV-2-specific IgA and IgG in human milk unlike convalescent women. **a** Concentration [pM^a^] of spike IgA and IgG in vaccinated women at T4 (*n* = 14) and T5 (*n* = 10) or in convalescent women (*n* = 6). **b** Concentration [pM^a^] of RBD IgA and IgG in vaccinated women at T4 (*n* = 14) and T5 (*n* = 10) or in convalescent women (*n* = 6). Each solid line represents one individual, and is colored to whether the IgA to IgA ratio was decreased (blue), or increased (red). Ratios were calculated by dividing the concentration of IgA over IgG antibodies. The red dotted line denotes the IgA detection limit, while the blue dotted line denotes the IgG detection limit in all assays. *p* values were calculated with Wilcoxon signed rank exact test, *p < 0.05, **p < 0.01, ***p < 0.001, n.s. non-significant. Box and whiskers plots (in gray) show median (center line), interquartile range (box), and 10th and 90th percentiles (whiskers). RBD receptor-binding domain, IgA immunoglobulin A, IgG immunoglobulin G, pM^a^ picomolar. ^a^SI conversion factors: to convert concentration from pM to M, multiply values by 10^12^.

However, to perform a direct cross-comparison between different antibody isotypes, OD_450_ values were further transformed to pM as the latter quantifies the absolute concentration of antibodies. Here, breaking down the analysis by individual women, we report a co-dominance of IgG and IgA at T4 with higher molar concentration of SARS-CoV-2-specific IgA compared to SARS-CoV-2-specific IgG in 57% of the women (8/14) for anti-spike and 43% of the women (6/14) for anti-RBD antibodies (Fig. 3). Due to the significant reduction in SARS-CoV-2-specific IgA at T5, 40% (4/10) of samples were observed to have a higher anti-spike IgA than IgG, while only 10% (1/10) were observed to have a higher anti-RBD IgA than IgG (Fig. 3). In the convalescent group, anti-spike IgG antibodies were only detected in one woman (Fig. 3a and Supplementary Fig. 4), and anti-RBD IgG antibodies were not detected in any sample (Fig. 3b and Supplementary Fig. 4).

### Minimal transfer of BNT162b2 mRNA in milk samples of vaccinated women

**Fig. 4:**
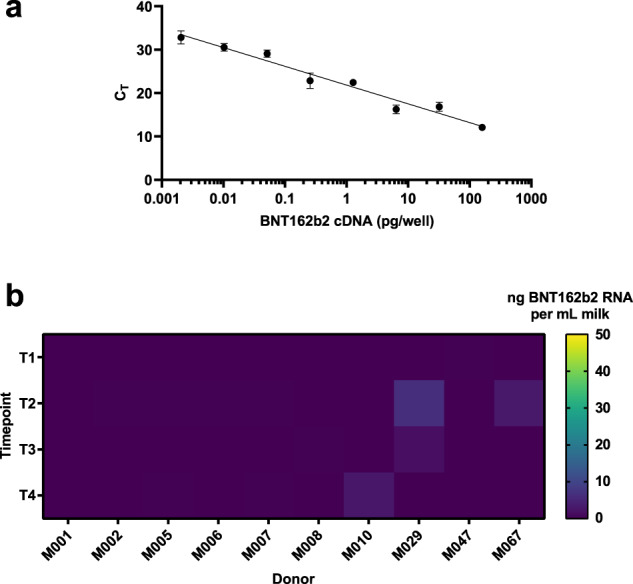
Minimal transfer of BNT162b2 mRNA into human milk. **a** Standard curve of cDNA reverse-transcribed from vaccine derived BNT162b2-spiked human milk was made and used as a positive control. *n* ≥ 6 technical replicates; means are shown with error bars indicating the standard error of measurement (SEM). **b** Heat map of calculated median BNT162b2 mRNA concentrations in vaccinated women (*n* = 10) at four time points as indicated. C_T_ cycle threshold, pg^a^ picogram, ng^b^ nanogram, mL^c^ milliliter, T1 pre-vaccination, T2 1–3 days after dose 1 of BNT162b2 vaccine, T3 7–10 days after dose 1 of BNT162b2 vaccine, T4 3–7 days after dose 2 of BNT162b2 vaccine. ^a^SI conversion factors: to convert concentration from pg to kg, multiply values by 10^15^. ^b^SI conversion factors: to convert concentration from ng to kg, multiply values by 10^12^. ^c^SI conversion factors: to convert concentration from ml to l, multiply values by 0.001.

There was minimal transfer of BNT162b2 mRNA into human milk across all time points. Our assay method could detect sub-picogram levels of BNT162b2 mRNA (Fig. 4a). The standard curves constructed with complementary DNA (cDNA) from spiked milk were similar to that obtained with a synthetic DNA construct (Supplementary Fig. 5), indicating robustness of the method. We were only able to observe on rare occasions very low levels of vaccine mRNA in human milk collected within the first week after either dose 1 or dose 2 (Fig. 4b); 36 out of 40 (90%) samples did not show detectable levels of vaccine mRNA. The highest concentration of BNT162b2 mRNA in the tested samples was 2 ng/ml. This would translate into a hypothetical 0.667% of the original vaccine dose being transferred in 100 ml of human milk given to the infant post-vaccination, in the worst-case scenario.

## Discussion

We demonstrated that human milk from lactating women who had received the BNT162b2 vaccine contained SARS-CoV-2-specific antibodies. Within 3–7 days after administering the second dose of BNT162b2 vaccine, 86% (12/14) of the individuals produced SARS-CoV-2-specific IgA, and 100% (14/14) individuals produced SARS-CoV-2-specific IgG in human milk. We observed sustained IgG responses to vaccination with no significant decrease out to 4–6 weeks post-second dose. While there was a reduction in the SARS-CoV-2-specific IgA response, anti-spike IgA was still detectable in 90% (9/10) of the individuals. The transfer of antibodies via milk may confer local mucosal protection to the breastfed infant. We detected negligible amounts of BNT162b2 mRNA in a minority of human milk samples using a very sensitive assay. Infants in our cohort had no reported adverse events up to 28 days after ingestion of postvaccination human milk.

We showed that the sharpest rise in antibody production was after dose 2 of the BNT162b2 vaccine, with a median of 827 pM of anti-spike IgA and 282 pM of anti-RBD IgA being seen in the human milk at T4 (3–7 days after dose 2). This is similar to the kinetics found in Baird et al. and Friedman et al., and could either be a function of time after the initial antigenic stimulation of dose 1, or an immunological need for dose 2^9,10^. As the humoral immunological response in lactating individuals has been recently shown^14^ to be more dependent on the booster dose of vaccination compared to non-lactating individuals, the latter scenario is more probable. Thus, whether significant amounts of antigen-specific antibodies can be elicited after administering one-dose regime vaccines, requires confirmation^14^.

Other groups have also found SARS-CoV-2 spike and RBD-specific IgG^9–12^ in human milk after vaccination in addition to SARS-CoV-2-specific IgA. Here, we showed that SARS-CoV-2 spike and RBD-specific IgG were found at significantly higher levels at T4 compared to prior timepoints. When comparing raw values at T4, a higher OD_450_ for SARS-CoV-2-specific IgG compared to SARS-CoV-2-specific IgA for spike and RBD was detected, suggesting that dominance of the SARS-CoV-2-specific IgG isotype might occur in BNT162b2 vaccinated women. Similar findings^9,10,15^ have been reported with relative quantification methods. However, when converted to pM, we show that the SARS-CoV-2-specific IgA and IgG levels are comparable and co-dominant at the population level. This demonstrates the importance of using absolute quantification in cross-comparing IgA and IgG levels.

Although SARS-CoV-2-specific IgA/IgG co-dominance was observed at both timepoints after the second dose (T4 and T5), we showed that the SARS-CoV-2-specific IgG response was more durable than that for SARS-CoV-2-specific IgA, with a decrease of IgA at T5 (4–6 weeks after dose 2). A faster decline in plasma IgA^16^ is known to occur in COVID-19 convalescent individuals, and reports of the same phenomenon^17^ are emerging for vaccinated individuals; the faster decline of IgA levels compared to IgG in our study may be related to the natural kinetics of IgA. Notably, convalescent women who had detectable antibodies in human milk (collected at a mean of 5.5 months post infection), had a higher level of SARS-CoV-2-specific IgA compared to IgG. The presence of vaccine-elicited IgG in human milk has been described after intramuscular influenza vaccination^3^ and is postulated to be related to the intramuscular route of antigenic exposure during vaccination. Compared to a mucosal exposure during natural infection, this would potentially induce increased class-switching to favor SARS-CoV-2-specific IgG as the dominant isotype rather than SARS-CoV-2-specific IgA in human milk^9^.

The role of antigen-specific IgG in human milk is unclear at this time, since IgG does not have a secretory chain and is prone to digestion by the breastfeeding infant; this lends voice to the move to develop intranasal vaccines for respiratory diseases^18^. The production of degradation-resistant spike-specific IgA in response to vaccination can be expected to confer mucosal immunity to the infant.

In terms of mRNA detection, it is worth noting that our method offers significant advantages over that reported previously^13^. Phenol-chloroform extraction, the gold standard for RNA extraction, and double-quencher qPCR probes were utilized in our protocol enhancing the sensitivity of our approach. Consequently, we could detect as low as 2 femtograms (2 × 10^–15^ g) of BNT162b2 cDNA input, which marks an ~60-fold increase in sensitivity relative to Golan et al.^13^, where they did not detect vaccine mRNA in human milk up to 48 h post-vaccination. However, translation and inferred persistence of nanoparticle-delivered mRNA on the scale of days have been previously reported in in vivo models^19^. Thus, we interrogated human milk for presence of mRNA up to 1 week post-vaccination in case of delayed kinetics. In addition, it is important to note that our protocol was designed for specific detection of intact BNT162b2 mRNA, rather than its degraded products, which would be more reflective of whole vaccine components.

Reassuringly, our data suggest that in most cases, vaccine mRNA does not escape into mammary secretions. The few instances where extremely low levels of BNT162b2 mRNA were detected may be due to naturally occurring inter-individual variations in protein adsorption^18^. This miniscule amount of mRNA is expected to be readily destroyed by enzymes in the infant’s gut, and any accompanying lipid nanoparticles that are excreted into human milk would also be readily digested if ingested orally by the infant^4,20^.

This study has several important strengths. Firstly, we accurately quantified antigen-specific IgA and IgG in human milk. This was referenced against a cohort of lactating women who are convalescent from antenatal COVID-19 and a cohort of unvaccinated lactating women. Secondly, we used a gold standard method for detection of vaccine mRNA in human milk, with actual BNT162b2 used as positive control. Lastly, we tracked and reported the clinical status of infants who were fed human milk from vaccinated women.

We acknowledge the limitations present in our study. Firstly, we did not perform any functional assays; however, spike- and RBD-specific antibody titers have been positively correlated with neutralization^21^. Secondly, several in the vaccinated cohort had antibodies prior to receiving the vaccine which suggests that these healthcare workers might have unknowingly acquired coronavirus infection(s) prior to vaccination. Lastly, this is a small and homogenous population of 14 women, and hence may not be generalizable to other groups of women. Longer and larger studies on safety may be warranted.

These results lend immunological and clinical evidence to the current recommendations of the ACOG, RCOG, and WHO that lactating individuals could continue breastfeeding in an uninterrupted manner after receiving COVID-19 mRNA vaccines^5,6,22^.

## Methods

### Clinical sample collection

We conducted a prospective cohort study of a convenience sample of lactating healthcare workers living in Singapore, who were due to receive two doses of the BNT162b2 (Pfizer/BioNtech) vaccine. The study was approved by the Domain Specific Review Board of the National Healthcare Group, Singapore (Gestational Immunity For Transfer GIFT-2: DSRB Reference Number: 2021/00095) and the study protocol was registered at clinicaltrials.gov (NCT04802278)^23^. Participants were invited to participate via advertisements and social media and recruited after informed consent was obtained, between 5 February 2021 and 9 February 2021. Exclusion criteria were that of no prior known exposure to COVID-19, and any autoimmune disease, current or recent infections, cancer, and any current or recent immunomodulatory medication. Demographic details and clinical outcomes of lactating women and their infants were determined through a structured questionnaire up to 28 days following the mother’s second dose of vaccine. Serum SARS-CoV-2 IgG were not assessed for mothers before the start of the study, due to the low community seroprevalence rate of 0.25% in Singapore^24^. Healthcare workers were also not deemed to be at significantly higher risk of SARS-CoV-2 transmission compared to the general population, as there were no outbreaks of Covid-19 clusters in healthcare institutions before the completion of the study.

Human milk samples from the “vaccinated cohort” were collected at five time points: pre-vaccination (T1), 1–3 days after dose 1 (T2), 7–10 days after dose 1 (T3), 3–7 days after dose 2 of COVID-19 mRNA vaccine (T4), and 4–6 weeks after dose 2 of COVID-19 mRNA vaccine (T5). A total of 14 vaccinated individuals were recruited. The samples from T1 to T4 were collected from all vaccinated individuals. Ten T5 samples were collected as the other four individuals have discontinued breastfeeding. All samples were collected at home using electric breast pumps or hand expressed into sterile plastic containers, stored, and transported via courier immediately at −18 °C to the laboratory. In the laboratory, the samples were stored at −80 °C until analysis.

Human milk samples at 1 month postpartum from convalescent and healthy women were also analyzed. The “convalescent cohort” were six women who had COVID-19 in pregnancy confirmed with real-time polymerase-chain-reaction (RT-PCR) assay, and then recovered as defined by resolution of clinical symptoms and with two negative RT-PCR assays 24 h apart. None had persistent symptoms suggestive of long COVID. PCR repeat per-partum as part of institutional protocol for convalescent COVID-19 mothers were all negative. Nine healthy unrelated women without COVID-19 infection or vaccination served as the “healthy cohort”.

Demographic details and clinical outcomes of lactating women and their infants were determined up to 28 days through a structured questionnaire after ingestion of post-vaccination human milk.

### Quantitative ELISA

IgA and IgG against SARS-CoV-2 antigens including the whole spike and RBD protein were quantified using ELISA. A human monoclonal antibody specific for SARS-CoV-2 was recombinantly engineered and expressed as human IgG1 and/or IgA1. This was employed as the reagent for quantitation.

Ninety-six-well flat-bottom maxi-binding immunoplate (SPL Life Sciences, #32296) were coated with 100 ng of SARS-CoV-2 whole spike protein or 200 ng of RBD protein at 4 °C overnight. After three washes in phosphate buffer saline (PBS), 350 µl of blocking buffer [4% skim milk in PBS with 0.05% Tween 20 (PBST)] was added to each well. After incubation for 1.5 h, the plate was washed three times with PBST. In total, 100 µl of ten-fold diluted human milk samples were added to each well for 1-h incubation. Plate was then washed three times with PBST followed by 1-h incubation in the dark with 100 µl of 5000-fold diluted goat anti-human IgG-HRP (Invitrogen, #31413), or 5000-fold diluted F(ab′)2 anti-human IgA-HRP (Invitrogen, #A24458). Plate was washed three times in PBST and incubated for 3 min with 1-Step Ultra TMB-ELISA (Thermo Scientific, #34029), 100 µl per well. Reaction was stopped with 100 µl of 1 M H_2_SO_4_ and OD_450_ was measured using a microplate reader (Tecan Sunrise). OD_450_ was calculated by subtracting the background signal from sample binding to the blocking buffer. A recombinant human monoclonal antibody targeting RBD of SARS-CoV-2 was engineered to both human IgG1 and human IgA1. Standard curves for whole spike and RBD were constructed by testing known concentrations of this antibody alongside the samples. To be specific, Human IgG1 was tested at 3.33, 6.67, 16.67, 33.33, 50, 66.67, 166.67, 333.33, 500, 666.67, and 1666.67 pM. Human IgA1 was tested at 6.67, 16.67, 33.33, 50, 66.67, 166.67, 333.33, 500, 666.67, 1666.67, and 3333.33 pM. Linear region of the standard curve was used to quantify the IgG/IgA antibody concentration in human milk samples via interpolation. Experiments were performed at least three times.

### mRNA extraction and quantification of BNT162b2 mRNA

Total ribonucleic acid (RNA) from whole human milk was extracted with TRIzol LS reagent (Invitrogen) according to manufacturer’s instructions. Briefly, 250 μl of whole human milk was RNA-extracted with 750 μl of Trizol LS and 200 μl of chloroform. The entire upper aqueous fraction (~500 μl) was isopropanol-precipitated, washed with 70% ethanol, and air-dried before dissolution in RNAse-free water. The entire volume of RNA was used as input for SuperScript IV (Invitrogen) reverse transcription performed as per manufacturer’s instructions and diluted 50-fold with RNAse-free water to a final volume of 1 ml prior to storage at −20 °C or assay. For standard curve construction, we collected BNT162b2 from actual vaccine discards. Five-fold serial dilutions of reconstituted BNT162b2 vaccine were spiked into healthy, SARS-CoV-2 negative human milk and RNA-extracted in parallel with vaccinee samples. These served as a positive control.

Taqman-based detection of BNT162b2 mRNA was performed using PrimeQuest-designed primer and probe sets (Integrated DNA Technologies (IDT)). The primer and probe sequences are as follows: AGCCTACACCAACAGCTTTAC (forward primer), TGAAGAAAGGCAGGAACAGG (reverse primer) and /56-FAM/CGACAAGGT/ZEN/GTTCAGATCCAGCGT/3IABkFQ/ (probe). Primers were used at 250 nM while probe was used at 150 nM. An input volume of 1 μl was used for each diluted cDNA sample. Standard cycling conditions used were as per recommendation by IDT.

A plasmid construct containing BNT162b2 cDNA without the 5′ and 3′ UTRs was synthesized (Twist Bioscience) and is herein referred to as BNT162b2∆UTR. This plasmid has been subcloned, sequence-verified, and made available through Addgene (ID #171214). Briefly, the BNT162b2 open reading frame is 3825 bases long and consists of human codon-optimized SARS-CoV-2 spike glycoprotein containing the K986P and V987P mutations^25^.

Standard curve construction was performed in a separate experiment using the BNT162b2ΔUTR construct and cDNA generated from BNT162b2-spiked human milk with the aim of determining whether their performances in the quantitative PCR assay were comparable.

### Statistics

Clinical characteristics of the cohorts were summarized using GraphPad Prism 8. Shapiro–Wilk test of normality was used; if data were normal, mean and SD were presented. The rest of the statistical analyses were done in R (4.0.2). Two groups were compared with Mann–Whitney *U* test (two tailed), or Wilcoxon signed rank exact test (paired) when indicated. For multiple comparisons, the PMCMRplus package (PMCMRplus_1.9.0) was used to perform Kruskal–Wallis test with Dunn’s nonparametric all-pairs comparison posttest. *p* < 0.05 level of confidence was accepted for statistical significance. Details of sample sizes and analyses performed specific to each figure are in all figure legends. All box and whiskers plots show median (center line), interquartile range (box), and 10th and 90th percentiles (whiskers). All data points are plotted with the boxplots. All line plots show the median, and error bars show the first and third quartiles. The study is reported in accordance to the STROBE reporting guidelines for cohort studies^26^.

### Reporting summary

Further information on research design is available in the Nature Research Reporting Summary linked to this article.

## Supplementary information

Supplementary Information

Reporting Summary

## Data Availability

The data are confidential and but can be shared in an anonymised manner upon request.
