# Supplementary material for: Codominant IgG and IgA expression with minimal vaccine mRNA in milk of BNT162b2 vaccinees
Source: NPJ Vaccines. 2021 Aug 19;6:105. doi: 10.1038/s41541-021-00370-z (PMC8376902; doi:10.1038/s41541-021-00370-z)
Supplement: Supplementary file 1 — Supplementary Information [file 41541_2021_370_MOESM1_ESM.pdf]

Supplementary Information

Supplementary Figures

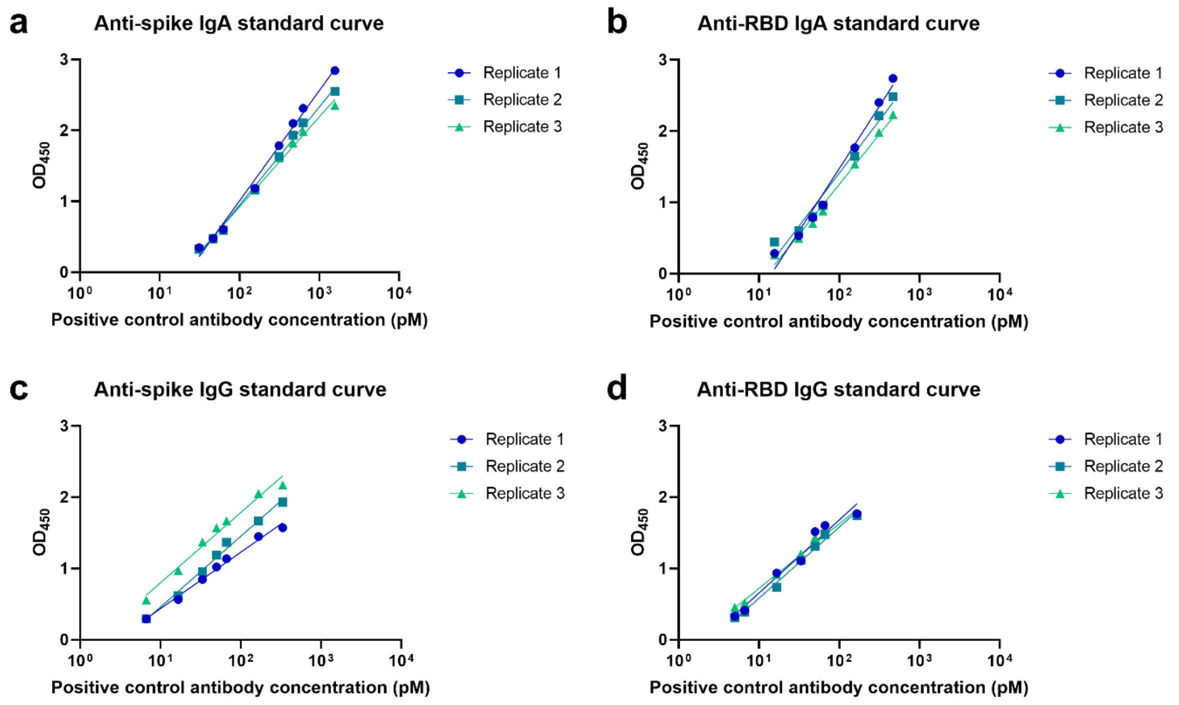

Supplementary Figure 1. Standard curves used in quantitative ELISA. A human monoclonal antibody binding to both full spike and RBD of SARS-CoV-2 was recombinantly expressed as human IgA1 and IgG1. Concentrations of **(a)** anti-spike IgA, **(b)** anti-RBD IgA, **(c)** anti-spike IgG, and **(d)** anti-RBD IgG antibodies in human milk samples were estimated from interpolation of the standard curves constructed using known concentrations of this antibody (in pM[a]).

Abbreviations: RBD, receptor-binding domain; IgA1, immunoglobulin A1; IgG1, immunoglobulin G1; pM, picomolar; OD_{450}, optical density measured at a wavelength of 450 nanometer.

[a] SI conversion factors: To convert concentration from pM to M, multiply values by 10^{12}.

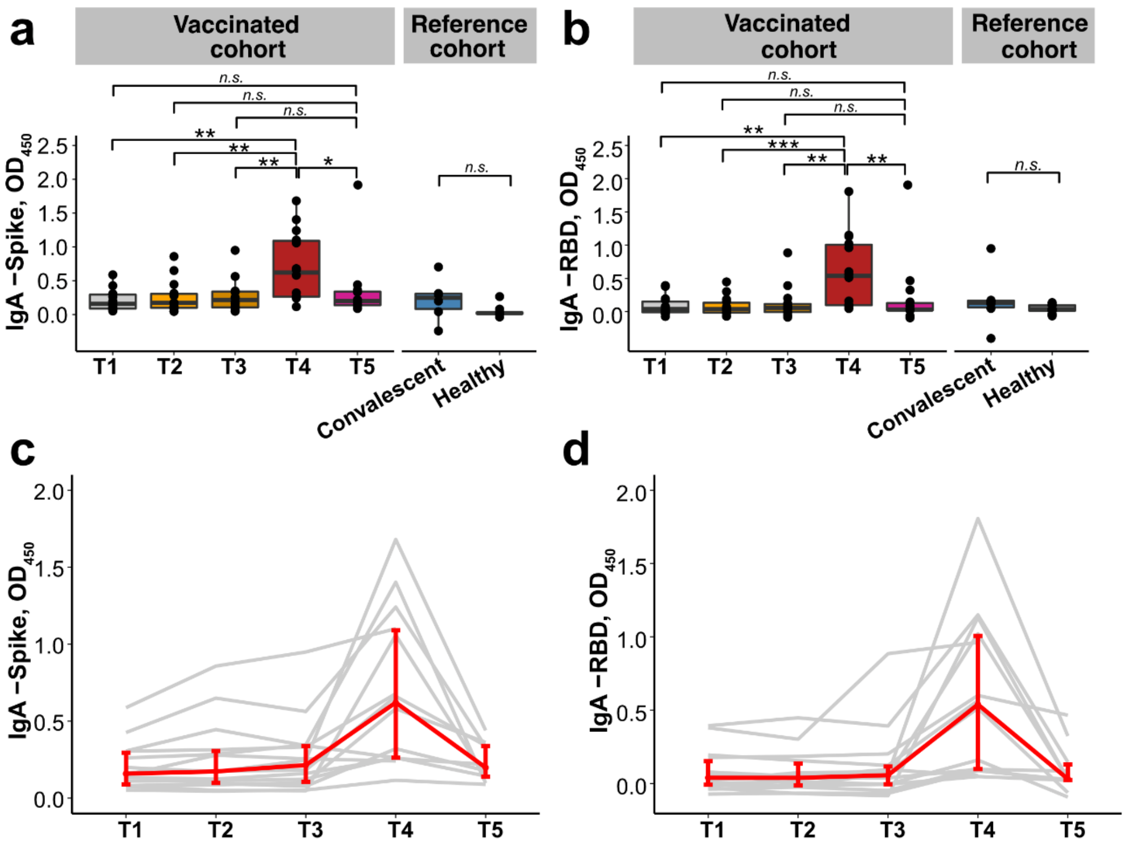

Supplementary Figure 2. SARS-CoV-2 antigen-specific IgA levels in human milk samples. OD_{450} values were measured for **(a)** anti-spike and **(b)** anti-RBD IgA antibodies in 10-fold diluted human milk samples collected from vaccinated women (n = 14) at T1-T4 timepoints and (n = 10) at T5 pre- and post- vaccination. Statistics were calculated with Kruskal-Wallis test with Dunn's post-test, *P<.05, **P<.01,***P<.001, n.s., non-significant. The same results are shown as line plots in **(c)** and **(d)** for visualization of antibody response from each individual. Each line in grey represents data from one individual and the median ± IQR is represented in red.

Abbreviations: OD_{450}, optical density measured at a wavelength of 450 nanometer; RBD, receptor-binding domain; IgA, immunoglobulin A; IQR, interquartile range; T1, pre-vaccination; T2, 1-3 days after dose one of BNT162b2 vaccine; T3, 7-10 days after dose one of BNT162b2 vaccine; T4, 3-7 days after dose two of BNT162b2 vaccine; T5, 4-6 weeks after dose two of BNT162b2 vaccine.

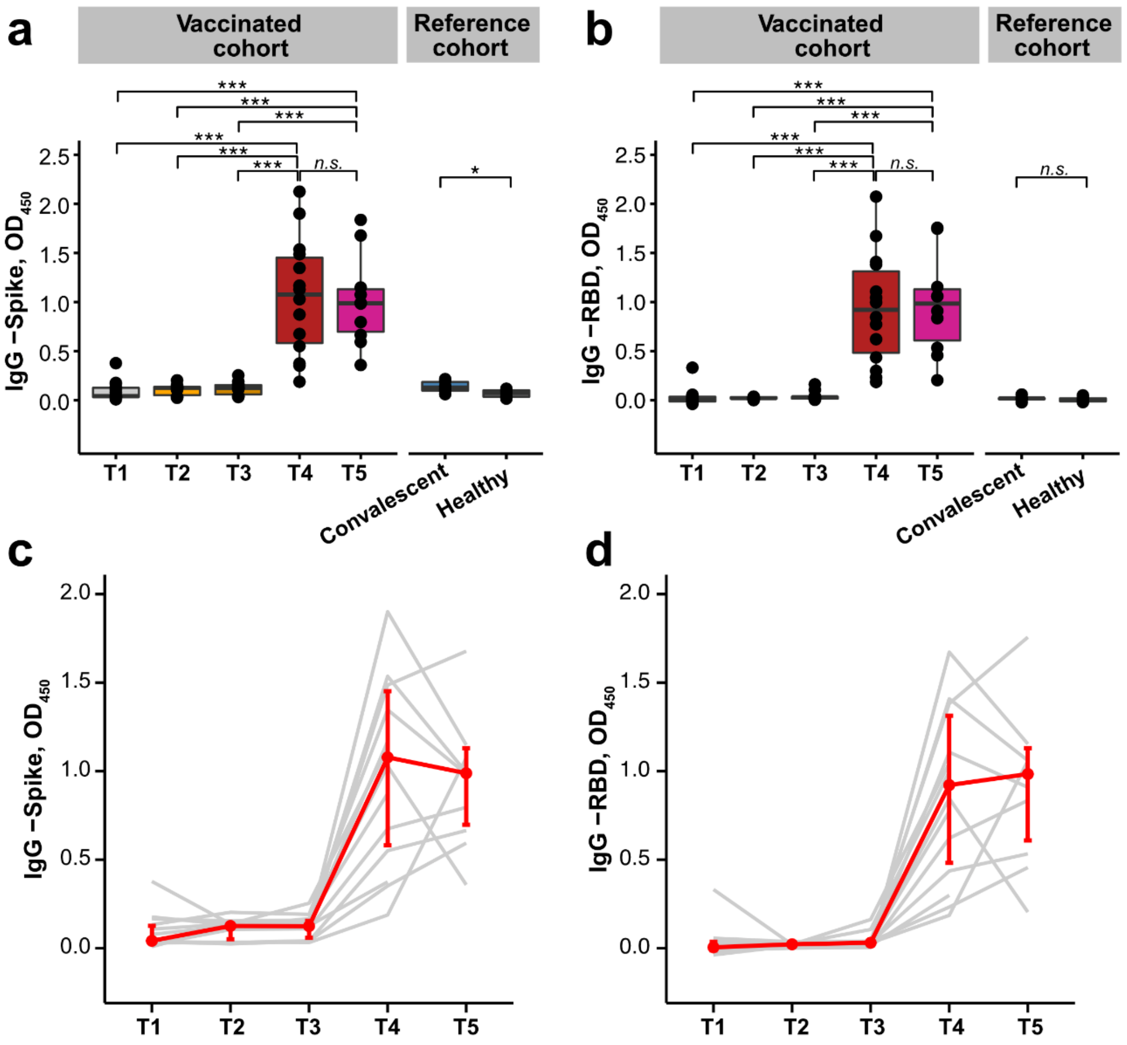

Supplementary Figure 3. SARS-CoV-2 antigen-specific IgG levels in human milk samples. OD_{450} values were measured for **(a)** anti-spike and **(b)** anti-RBD IgG antibodies in 10-fold diluted human milk samples collected from vaccinated women (n = 14) at T1-T4 timepoints and (n = 10) at T5 pre- and post- vaccination. Statistics were calculated with Kruskal-Wallis test with Dunn's post-test, *P<.05, **P<.01,***P<.001, n.s., non-significant. The same results are shown as line plots in **(c)** and **(d)** for visualization of antibody response from each individual. Each line in grey represents data from one individual and the median ± IQR is represented in red.

Abbreviations: OD_{450}, optical density measured at a wavelength of 450 nanometer; RBD, receptor-binding domain; IgG, immunoglobulin G; IQR, interquartile range; T1, pre-vaccination; T2, 1-3 days after dose one of BNT162b2 vaccine; T3, 7-10 days after dose one

of BNT162b2 vaccine; T4, 3-7 days after dose two of BNT162b2 vaccine; T5, 4-6 weeks after dose two of BNT162b2 vaccine.

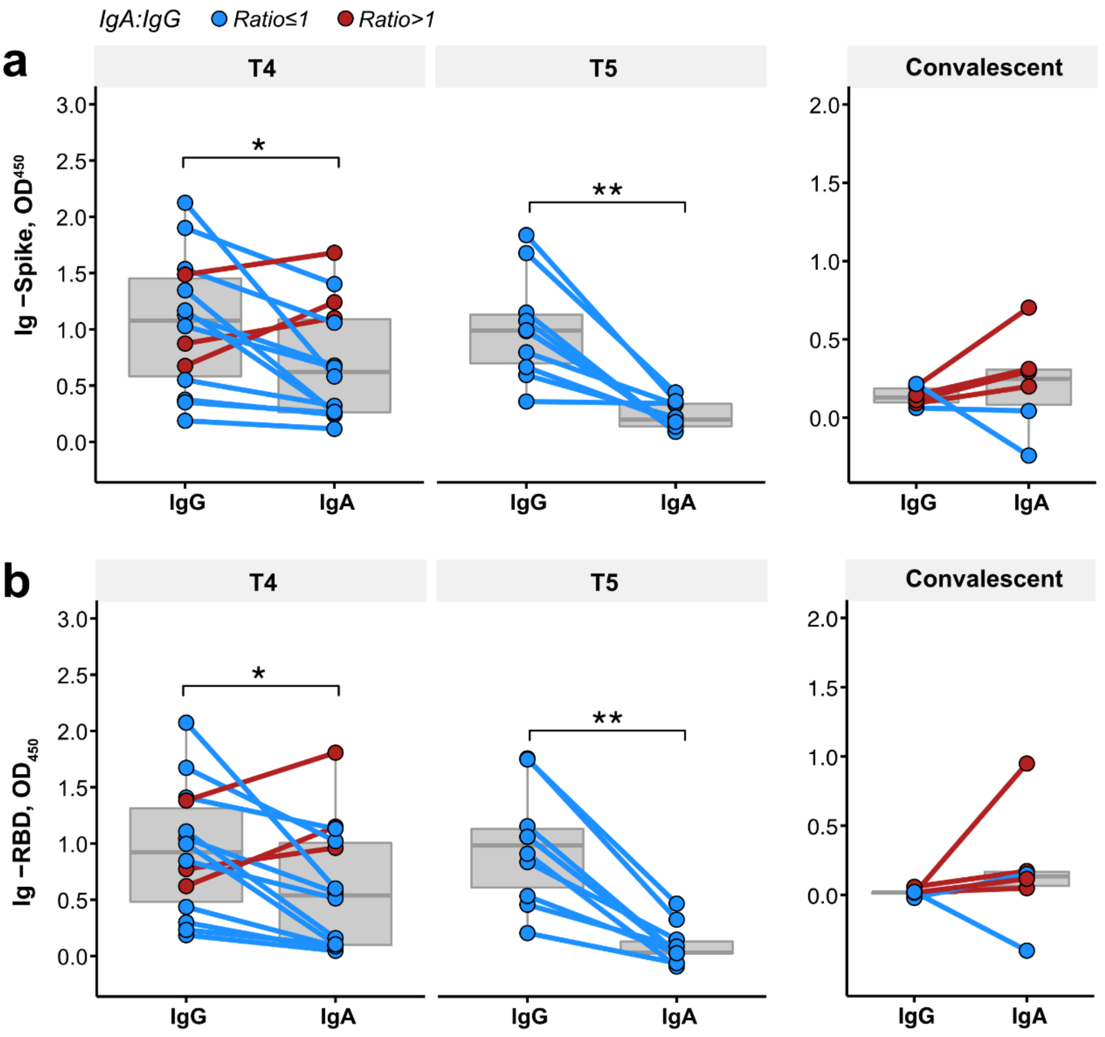

Supplementary Figure 4. Cross-comparison of SARS-CoV-2 antigen-specific IgA and IgG levels in human milk samples. (a) OD_{450} values were measured for vaccinated women at T4 (n = 14) and T5 (n = 10) or in convalescent women (right, n = 6) and show anti-spike in 10-fold diluted human milk samples. **(b)** OD_{450} values were measured for vaccinated women at T4 (n = 14) and T5 (n = 10) or in convalescent women (right, n = 6) and show anti-RBD in 10-fold diluted human milk samples. Each solid line represents one individual, and is colored to whether the IgA to IgA ratio was decreased (blue), or increased (red). Ratios were calculated by dividing the concentration of IgA over IgG antibodies. P values were calculated with Wilcoxon signed rank exact test, $^*P<0.05$, $^{**}P<0.01$.

Abbreviations: OD_{450}, optical density measured at a wavelength of 450 nanometer; RBD, receptor-binding domain; IgA, immunoglobulin A; IgG, immunoglobulin G; T4, 3-7 days after dose two of BNT162b2 vaccine; T5, 4-6 weeks after dose two of BNT162b2 vaccine.

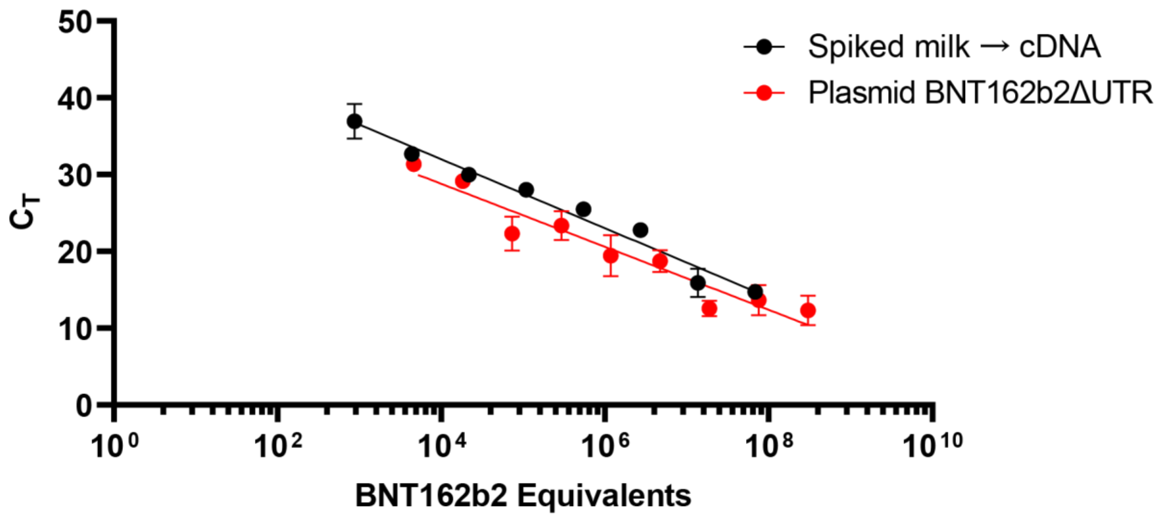

Supplementary Figure 5. Standard curves using cDNA generated from BNT162b2-spiked human milk versus pTwist-BNT162b2ΔUTR plasmid. One BNT162b2 equivalent is defined as a single-stranded polynucleotide molecule that contains a linear sequence that binds either the forward or reverse primer. Prism analysis of the two regression lines revealed that their slopes/gradients were not significantly different ($P = .42$). Data represented as the mean ± SEM, n ≥ 3 technical replicates.

Abbreviations: cDNA, complementary DNA; C_T, cycle threshold.

Supplementary Table 1: Demographic and clinical details of infants born to mothers in convalescent and control cohorts

**Demographic and clinical details of infants**	**Convalescent cohort (n = 6)**	**Control cohort (n = 9)**
Mode of delivery	6 Vaginal	6 Vaginal
		3 Caesarean section
Gender of infants	5 Female	6 Female
Infant birth weight, mean [SD], kg	3.59 [0.24]	3.11 [0.61]
Infant birth length, mean [SD], cm	50.8 [1.6]	50.5 [2.2]
Infant birth occipital frontal circumference, mean [SD], cm	34.6 [0.9]	22.3 [1.2]
Any immediate postnatal complications	5 None	8 None
	1 Transient tachypnoea of the newborn	1 Exaggerated physiological jaundice

Supplementary Table 2: Clinical details of antenatal COVID-19 and perinatal findings for convalescent cohort (n = 6)

**Clinical details of antenatal COVID-19 and perinatal findings**	
COVID-19 diagnosis gestation	2 First trimester<br>1 Second trimester<br>3 Third trimester
Length of time between COVID-19 diagnosis and delivery, mean [SD], days	136 [80]
Severity of COVID-19	1 Asymptomatic<br>4 Mild<br>1 Moderate
Time between first positive PCR and first negative PCR, median (range), days	23 (17 - 39)
SARS-CoV-2 PCR from perinatal samples (nasopharyngeal, vaginal, umbilical cord and placental swabs, maternal blood, cord blood, amniotic fluid, human milk)	All negative
Placental / umbilical cord histology	2 Normal<br>3 Abnormal<br>1 Not done
Abbreviations: COVID-19, coronavirus disease of 2019; PCR, polymerase chain reaction	

Supplementary Spreadsheet 1

**Spike (pM)**	T1	T2	T3	T4
T2	0.348759784	NA	NA	NA
T3	0.490751122	0.804224959	NA	NA
T4	**7.77426E-05**	**0.002576096**	**0.001105746**	NA
T5	**0.006952115**	**0.065216457**	**0.038447045**	*0.3639543*

	T1	T2	T3	T4
T2	*n.s.*	NA	NA	NA
T3	*n.s.*	*n.s.*	NA	NA
T4	***	**	**	NA
T5	**	**0.06521646**	*	*n.s.*

**RBD (pM)**	T1	T2	T3	T4
T2	0.682652666	NA	NA	NA
T3	0.964745325	0.715375337	NA	NA
T4	**0.005933693**	**0.001576234**	**0.005179982**	NA
T5	0.99356138	0.714996495	0.974249714	**0.01174336**

	T1	T2	T3	T4
T2	*n.s.*	NA	NA	NA
T3	*n.s.*	*n.s.*	NA	NA
T4	**	**	**	NA
T5	*n.s.*	*n.s.*	*n.s.*	*

**Spike (OD450)**	T1	T2	T3	T4
T2	0.686483005	NA	NA	NA
T3	0.561350337	0.859347762	NA	NA
T4	**0.001159137**	**0.004439516**	**0.007632421**	NA
T5	0.448153305	0.696512151	0.819437778	**0.0273007**

	T1	T2	T3	T4
T2	*n.s.*	NA	NA	NA
T3	*n.s.*	*n.s.*	NA	NA
T4	**	**	**	NA
T5	*n.s.*	*n.s.*	*n.s.*	*

**RBD (OD450)**	T1	T2	T3	T4
T2	0.867085576	NA	NA	NA
T3	0.832369848	0.70466953	NA	NA
T4	**0.00130751**	**0.00072042**	**0.002676285**	NA
T5	0.9863765	0.865124289	0.860179423	**0.00353201**

	T1	T2	T3	T4
T2	*n.s.*	NA	NA	NA
T3	*n.s.*	*n.s.*	NA	NA
T4	**	***	**	NA
T5	*n.s.*	*n.s.*	*n.s.*	**

Kruskal-wallis with Dunn's post test

*	p<0.05
**	p<0.01
***	p<0.001
n.s.	p>0.05

	p value	
**IgA - Spike (pM)**	0.02850533	*
**IgA - RBD (pM)**	0.3541089	*n.s.*
**IgA - Spike (OD450)**	**0.08791209**	*n.s.*
**IgA - RBD (OD450)**	0.1134865	*n.s.*
**IgG - Spike (pM)**	0.7650872	*n.s.*
**IgG - RBD (pM)**	0.5286713	*n.s.*
**IgG - Spike (OD450)**	0.03596404	*
**IgG - RBD (OD450)**	NA	*n.s.*

Mann Whitney test comparing convalescent and

*	p<0.05
**	p<0.01
***	p<0.001
n.s.	p>0.05

**Spike (OD45**	T1	T2	T3	T4
T2	0.53510723	NA	NA	NA
T3	0.31767005	0.70466659	NA	NA
T4	**4.52E-07**	**9.63E-06**	**5.20E-05**	NA
T5	**5.09E-06**	**6.48E-05**	**0.0002635**	*0.9641586*

	T1	T2	T3	T4
T2	*n.s.*	NA	NA	NA
T3	*n.s.*	*n.s.*	NA	NA
T4	***	***	***	NA
T5	***	***	***	*n.s.*

**RBD (OD450**	T1	T2	T3	T4
T2	0.62952891	NA	NA	NA
T3	0.24939125	0.50321534	NA	NA
T4	**6.00E-07**	**6.52E-06**	**0.00012332**	NA
T5	**2.60E-06**	**2.05E-05**	**0.00026358**	*0.8856655*

	T1	T2	T3	T4
T2	*n.s.*	NA	NA	NA
T3	*n.s.*	*n.s.*	NA	NA
T4	***	***	***	NA
T5	***	***	***	*n.s.*

**Spike (pM)**	T1	T2	T3	T4
T2	0.50662712	NA	NA	NA
T3	0.31538257	0.73393707	NA	NA
T4	**3.24E-07**	**8.80E-06**	**4.05E-05**	NA
T5	**3.28E-07**	**6.78E-06**	**2.79E-05**	*0.6577786*

	T1	T2	T3	T4
T2	*n.s.*	NA	NA	NA
T3	*n.s.*	*n.s.*	NA	NA
T4	***	***	***	NA
T5	***	***	***	*n.s.*

**RBD (pM)**	T1	T2	T3	T4
T2	0.77952923	NA	NA	NA
T3	0.77952923	1	NA	NA
T4	**6.12E-07**	**1.38E-07**	**1.38E-07**	NA
T5	**2.44E-05**	**7.62E-06**	**7.62E-06**	*0.7393407*

	T1	T2	T3	T4
T2	*n.s.*	NA	NA	NA
T3	*n.s.*	*n.s.*	NA	NA
T4	***	***	***	NA
T5	***	***	***	*n.s.*

Kruskal-wallis with Dunn's post test

*	p<0.05
**	p<0.01
***	p<0.001
n.s.	p>0.05

**Spike (pM)**		
T4	0.2676	*n.s.*
T5	0.1602	*n.s.*
Convalescent	0.1814	*n.s.*

**RBD (pM)**		
T4	1	*n.s.*
T5	0.01285	*
Convalescent	0.3711	*n.s.*

**Spike (OD450)**		
T4	0.04187	*
T5	0.001953	**
Convalescent	0.4375	*n.s.*

**RBD (OD450)**		
T4	0.04944	*
T5	0.001953	**
Convalescent	0.3125	*n.s.*

Wilcox signed rank exact test comparin

*	$p<0.05$
**	$p<0.01$
***	$p<0.001$
n.s.	$p>0.05$

Supplementary Spreadsheet 2

IgA - Spike,pM

Timepoint	N	Mean	Median	Q1	Q3	IQR
T1	14	144.9365193	111.220315	0	218.153724	218.153724
T2	14	255.2765672	234.473256	27.0021855	341.867559	314.865373
T3	14	234.8559409	117.857385	27.102247	284.795295	257.693048
T4	14	1499.672907	826.835075	571.860637	1470.26792	898.407281
T5	10	476.8536117	498.571931	387.887983	635.426631	247.538648

IgA - RBD,pM

Timepoint	N	Mean	Median	Q1	Q3	IQR
T1	14	46.969842	0	0	59.5702703	59.5702703
T2	14	39.58761936	0	0	38.6442398	38.6442398
T3	14	57.30281571	0	0	52.6867318	52.6867318
T4	14	463.3496039	281.515753	13.4201603	555.751253	542.331093
T5	10	74.3794641	0	0	124.887315	124.887315

IgA - Spike, OD450

Timepoint	N	Mean	Median	Q1	Q3	IQR
T1	14	0.209347619	0.1585	0.08824167	0.29425	0.20600833
T2	14	0.263254762	0.173	0.09880833	0.306	0.20719167
T3	14	0.269661905	0.21433333	0.10611667	0.33866667	0.23255
T4	14	0.704704762	0.62081667	0.26266667	1.09025	0.82758333
T5	10	0.233683333	0.19815	0.1384	0.338475	0.200075

IgA - RBD,OD450

Timepoint	N	Mean	Median	Q1	Q3	IQR
T1	14	0.088419048	0.03866667	-0.00915	0.15194167	0.16109167
T2	14	0.079647619	0.03788333	-0.0136167	0.13508333	0.1487
T3	14	0.122345238	0.05616667	-0.005975	0.11575	0.121725
T4	14	0.592426191	0.53866667	0.0981	1.00616667	0.90806667
T5	10	0.09707	0.03021667	0.02248333	0.13036667	0.10788333

IgG - Spike,pM

	Timepoint	N	Mean	Median	Q1	Q3	IQR
1	T1	14	2.319225286	0	0	0	0
2	T2	14	14.62996807	0	0	20.0292023	20.0292023
3	T3	14	21.05971486	0	0	33.742577	33.742577
4	T4	14	915.3158191	392.378148	144.46501	1000.73262	856.267606
5	T5	10	1422.407878	657.405417	264.878147	1227.69151	962.813361

IgG - RBD,pM

	Timepoint	N	Mean	Median	Q1	Q3	IQR
1	T1	14	3.214945143	0	0	0	0
2	T2	14	0	0	0	0	0
3	T3	14	0	0	0	0	0
4	T4	14	373.0752676	188.458432	68.7087688	501.146096	432.437327
5	T5	10	286.0967628	184.11296	89.8310483	250.409877	160.578829

IgG - Spike, OD450

	Timepoint	N	Mean	Median	Q1	Q3	IQR
1	T1	14	0.091647619	0.04171667	0.03235	0.12641667	0.09406667
2	T2	14	0.106328571	0.12585	0.05123333	0.13575833	0.084525
3	T3	14	0.119116667	0.12486667	0.058775	0.1544	0.095625
4	T4	14	1.052276191	1.07816667	0.58171667	1.45166667	0.86995
5	T5	10	1.012836667	0.98831667	0.69756667	1.130275	0.43270833

IgG - RBD,OD450

	Timepoint	N	Mean	Median	Q1	Q3	IQR
1	T1	14	0.030611905	0.00516667	-0.0118333	0.03589167	0.047725
2	T2	14	0.021652381	0.0222	0.01603333	0.03005833	0.014025
3	T3	14	0.04025	0.03108333	0.01589167	0.03633333	0.02044167
4	T4	14	0.934266667	0.9215	0.48248333	1.31333333	0.83085
5	T5	10	0.971336667	0.98383333	0.60866667	1.13031667	0.52165
