# Supplementary material for: Codominant IgG and IgA expression with minimal vaccine mRNA in milk of BNT162b2 vaccinees
Source: NPJ Vaccines. 2021 Aug 19;6:105. doi: 10.1038/s41541-021-00370-z (PMC8376902; doi:10.1038/s41541-021-00370-z)
Supplement: Supplementary file 2 — Reporting Summary [file 41541_2021_370_MOESM2_ESM.pdf]

nature research

Corresponding author(s): *Double-blind peer review submissions: write DBPR and your manuscript number here instead of author names.*

Last updated by author(s): *YYYY-MM-DD*

Reporting Summary

Nature Research wishes to improve the reproducibility of the work that we publish. This form provides structure for consistency and transparency in reporting. For further information on Nature Research policies, see our Editorial Policies and the Editorial Policy Checklist.

Statistics

For all statistical analyses, confirm that the following items are present in the figure legend, table legend, main text, or Methods section.

n/a	Confirmed	
☐	☒	The exact sample size (*n*) for each experimental group/condition, given as a discrete number and unit of measurement
☐	☒	A statement on whether measurements were taken from distinct samples or whether the same sample was measured repeatedly
☐	☒	The statistical test(s) used AND whether they are one- or two-sided<br>*Only common tests should be described solely by name; describe more complex techniques in the Methods section.*
☐	☒	A description of all covariates tested
☐	☒	A description of any assumptions or corrections, such as tests of normality and adjustment for multiple comparisons
☐	☒	A full description of the statistical parameters including central tendency (e.g. means) or other basic estimates (e.g. regression coefficient) AND variation (e.g. standard deviation) or associated estimates of uncertainty (e.g. confidence intervals)
☐	☒	For null hypothesis testing, the test statistic (e.g. *F*, *t*, *r*) with confidence intervals, effect sizes, degrees of freedom and *P* value noted<br>*Give P values as exact values whenever suitable.*
☒	☐	For Bayesian analysis, information on the choice of priors and Markov chain Monte Carlo settings
☒	☐	For hierarchical and complex designs, identification of the appropriate level for tests and full reporting of outcomes
☒	☐	Estimates of effect sizes (e.g. Cohen's *d*, Pearson's *r*), indicating how they were calculated

Our web collection on statistics for biologists contains articles on many of the points above.

Software and code

Policy information about availability of computer code

Data collection	No software was used for data collection
Data analysis	GraphPad Prism 8, R (4.0.2) using base packages for Mann-Whitney U test (two sided), PMCMRplus (PMCMRplus_1.9.0) used for Kruskal-Wallis test with Dunn's nonparametric all-pairs comparison post-test.

For manuscripts utilizing custom algorithms or software that are central to the research but not yet described in published literature, software must be made available to editors and reviewers. We strongly encourage code deposition in a community repository (e.g. GitHub). See the Nature Research guidelines for submitting code & software for further information.

Data

Policy information about availability of data

All manuscripts must include a data availability statement. This statement should provide the following information, where applicable:

- Accession codes, unique identifiers, or web links for publicly available datasets
- A list of figures that have associated raw data
- A description of any restrictions on data availability

The data that support the findings of this study are available on request from the corresponding author (LWW). The data are not publicly available due to the presence of identifiers that could compromise research participant consent.

Field-specific reporting

Please select the one below that is the best fit for your research. If you are not sure, read the appropriate sections before making your selection.

☒ Life sciences ☐ Behavioural & social sciences ☐ Ecological, evolutionary & environmental sciences

For a reference copy of the document with all sections, see nature.com/documents/nr-reporting-summary-flat.pdf

Life sciences study design

All studies must disclose on these points even when the disclosure is negative.

Sample size	All sample sizes are indicated for each result figure. The sample size for the reference cohort was limited by the number of convalescent and healthy mothers that consented to the study. The sample sizes for the vaccinated cohort was determined to be adequate on the basis of the magnitude and consistency of measurable differences between groups.
Data exclusions	No data was excluded from the analyses
Replication	ELISAs for binding activity of IgA and IgG antibodies were performed with at least three technical replicates and results reflect three independent experiments. Quantitative PCR for BNT162b2 mRNA were performed with at least three technical replicates and results reflect three independent experiments.
Randomization	Due to the nature of a cohort study, the participants were not randomized to different groups.
Blinding	Investigators were not blinded to groups since the cohort was collected sequentially

Reporting for specific materials, systems and methods

We require information from authors about some types of materials, experimental systems and methods used in many studies. Here, indicate whether each material, system or method listed is relevant to your study. If you are not sure if a list item applies to your research, read the appropriate section before selecting a response.

Materials & experimental systems

n/a	Involved in the study	
☐	☒	Antibodies
☒	☐	Eukaryotic cell lines
☒	☐	Palaeontology and archaeology
☒	☐	Animals and other organisms
☐	☒	Human research participants
☐	☒	Clinical data
☒	☐	Dual use research of concern

Methods

n/a	Involved in the study	
☒	☐	ChIP-seq
☒	☐	Flow cytometry
☒	☐	MRI-based neuroimaging

Antibodies

Antibodies used	LSI-CoV2-PC1-IgG1: produced in-house at Antibody Engineering Programme, Life Science Institute, National University of Singapore. Lot #03.<br>LSI-CoV2-PC1-IgA1: produced in-house at Antibody Engineering Programme, Life Science Institute, National University of Singapore. Lot #01.<br>Goat anti-human IgG Fc Cross-Absorbed Secondary Antibody, HRP: Invitrogen, #31413<br>F(ab')2-Goat anti-Human IgA Secondary Antibody, HRP: Invitrogen, #A24458
Validation	LSI-CoV2-PC1-IgG1 is a recombinant antibody produced from a plasmid with human IgG1 constant region sequences. Monoclonality was confirmed by sequencing. Antibody isotype and antibody reactivity to target antigens SARS-CoV-2 spike and receptor-binding domain were confirmed by ELISA using secondary antibody goat anti-human IgG Fc, HRP (Invitrogen #31413).<br>LSI-CoV2-PC1-IgA1 is a recombinant antibody produced from a plasmid with human IgA1 constant region sequences. Monoclonality was confirmed by sequencing. Antibody isotype and antibody reactivity to target antigens SARS-CoV-2 spike and receptor-binding domain were confirmed by ELISA using secondary antibody F(ab')2-goat anti-human IgA, HRP (Invitrogen #A24458).

Human research participants

Policy information about studies involving human research participants

Population characteristics	Lactating women were prospectively recruited from Singapore when they had appointments to receive the first and second dose of mRNA SARS-CoV-2 vaccination; at the point of time that the study was conducted, only BNT162b2 was offered as a

	vaccine against COVID-19 in Singapore. Exclusion criteria were: no prior known exposure to COVID-19, autoimmune disease, current or recent infections, cancer or any current or recent immunomodulatory medication. Women of all ages, ethnicities, other co-morbidities, antenatal conditions and duration of time post-partum were included.
Recruitment	Participants were healthcare workers conveniently recruited from the National University Hospital, Singapore, via advertisements and social media. The reason why only healthcare workers could be recruited was because SARS-CoV-2 vaccination was only made available to this population. As the median age of recruited population was similar to the childbearing age of women in Singapore, with a similar ethnic distribution, we expect that this population is representative of the general lactating population.
Ethics oversight	National Healthcare Group Domain Specific Research Board (DSRB), Singapore. Reference number 2021/00095

Note that full information on the approval of the study protocol must also be provided in the manuscript.

Clinical data

Policy information about clinical studies

All manuscripts should comply with the ICMJE guidelines for publication of clinical research and a completed CONSORT checklist must be included with all submissions.

Clinical trial registration	NCT04802278
Study protocol	Study protocol is available on the DSRB portal and can be made available to all interested parties upon request.
Data collection	Subjects were recruited between 5 and 9 February 2021, and clinical data collected via a standardized online questionnaire.
Outcomes	The only clinical outcome measure determined for infants in this study was that of infant health after being fed post-vaccination milk. This was obtained by self-reporting of their mothers through the online questionnaire.
